# Fast photodynamics of azobenzene probed by scanning excited-state potential energy surfaces using slow spectroscopy

**DOI:** 10.1038/ncomms6860

**Published:** 2015-01-06

**Authors:** Eric M. M. Tan, Saeed Amirjalayer, Szymon Smolarek, Alexander Vdovin, Francesco Zerbetto, Wybren Jan Buma

**Affiliations:** 1 Van ’t Hoff Institute for Molecular Sciences, University of Amsterdam, Science Park 904, 1098 XH Amsterdam, The Netherlands; 2 Dipartimento di Chimica ‘G. Ciamician’, Università di Bologna, via F. Selmi 2, I-40126 Bologna, Italy

## Abstract

Azobenzene, a versatile and polymorphic molecule, has been extensively and successfully used for photoswitching applications. The debate over its photoisomerization mechanism leveraged on the computational scrutiny with ever-increasing levels of theory. However, the most resolved absorption spectrum for the transition to S_1_(nπ*) has not followed the computational advances and is more than half a century old. Here, using jet-cooled molecular beam and multiphoton ionization techniques we report the first high-resolution spectra of S_1_(nπ*) and S_2_(ππ*). The photophysical characterization reveals directly the structural changes upon excitation and the timescales of dynamical processes. For S_1_(nπ*), we find that changes in the hybridization of the nitrogen atoms are the driving force that triggers isomerization. In combination with quantum chemical calculations we conclude that photoisomerization occurs along an inversion-assisted torsional pathway with a barrier of ~2 kcal mol^−1^. This methodology can be extended to photoresponsive molecular systems so far deemed non-accessible to high-resolution spectroscopy.

## Abstract (web summary)

Azobenzene is perhaps the archetypal light-activated molecule, widely used for photoswitching applications, but the mechanism of isomerisation remains in doubt. Here, the authors provide high-resolution excitation spectra of trans-azobenzene, identifying the structural changes accompanying photoisomerisation.

## Graphical abstract

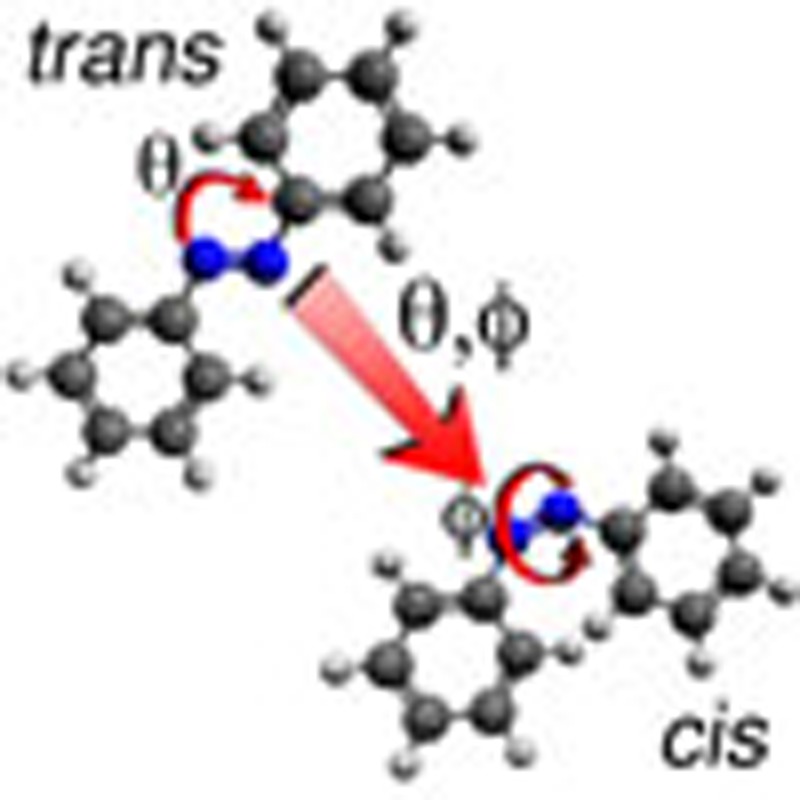

Light offers unique opportunities to address molecular systems non-invasively, in a highly selective manner, and with high space and time resolution. Compounds whose shape, biological and chemical activity, dielectric constant and refractive index can be switched reversibly under the control of light thus have found extensive applications in areas that range from molecular probes to molecular nanotechnology and photopharmacology^1^,^2^,^3^,^4^. Azobenzene and its derivatives are one of the primary examples of such compounds. Their favourable properties such as photostability and functional activity under a wide range of experimental conditions have in many aspects paved the way for the further development and application of photochromic systems.

At the basis of the functional behaviour of azobenzene is the reversible *trans* (E)→*cis* (Z) isomerization of the N=N bond upon photoexcitation. The absorption spectrum of azobenzene has therefore long attracted attention. Its low-energy part displays two bands, a weak band in the visible region (~430 nm in *n*-hexane) associated with excitation of the formally dipole-forbidden ^1^B_2g_(nπ*) state and a strong band in the ultraviolet region (~320 nm in *n*-hexane) associated with absorption of the dipole-allowed ^1^B_1u_(ππ*) state^5^. One of the intriguing aspects of azobenzene photoisomerization is the dependence of the quantum yield on the excitation wavelength^6^,^7^. In principle, isomerization in azobenzene can take place along two different pathways, one that involves torsional motion around the N=N double bond, and another in which in-plane inversion about one of the nitrogen centres takes place (Fig. 1). To accommodate the observed violation of Kasha’s rule, it was therefore originally proposed that isomerization proceeds along two different pathways in the nπ* and ππ* states^5^,^8^,^9^. Over the years, extensive experimental^10^,^11^,^12^,^13^,^14^,^15^,^16^,^17^,^18^,^19^ and theoretical^20^,^21^,^22^,^23^,^24^,^25^,^26^,^27^,^28^,^29^ efforts have been made to elucidate the contribution of these pathways to the photoisomerization dynamics. As yet, however, there is still no consensus on the pathway, and also the interpretation of many other aspects of the excited-state dynamics are still a matter of extensive debate.

At the basis of a fundamental understanding of the photodynamics of azobenzene is a detailed knowledge on the potential energy surfaces of the relevant excited states, preferably under isolated molecule conditions. Such studies provide detailed insight on the forces that act on the molecular structure after excitation from the ground state, map out which normal coordinates are involved in the structural dynamics, reveal whether energy barriers are involved and provide a direct benchmark for theoretical calculations^30^,^31^,^32^,^33^. Moreover, they must necessarily be the starting point for a further appreciation of the experimentally observed dependence of the photodynamics on the environment and substituents. As yet, however, azobenzene has predominantly been studied in the time domain with ultrafast laser techniques, at room temperature, and under non-isolated conditions. Under such conditions, temporal resolution is achieved at the expense of spectral resolution. As a result, the vibrational level structure of the excited state—which provides a direct fingerprint of the potential energy surface along the various normal coordinates—is not accessible.

Here, we show that advanced high-resolution two- and three-colour multiphoton ionization laser spectroscopic techniques using nanosecond lasers and molecular beams can be successfully applied to scan the potential energy surfaces of the S_1_(nπ*) and S_2_(ππ*) states of *trans*-azobenzene and measure the vibrational structure. Such an approach is well established, but its application to azobenzene appears to contradict *a priori* the conditions under which such studies are normally performed. Excitation of the S_1_(nπ*) is formally dipole-forbidden, and only becomes allowed when the state is vibronically coupled to another dipole-allowed excited state. In combination with an excited-state lifetime that has been reported to be in the low-picosecond regime^3^, resonance enhancement of the ionization yield is thus expected to be very hard to detect. Excitation of the S_2_(ππ*) state is fully allowed, but for this state the reported lifetime of ~170 fs in the gas phase^9^ severely impedes observation of resonance enhancement, and, if observed at all, lifetime broadening of resonances would be taken to preclude the detection of vibrationally resolved spectra. Despite these unfavourable conditions, we find that high-resolution spectra of both states can be obtained, and that these spectra provide a direct view on the photophysics and photochemistry of *trans*-azobenzene.

## Results

### Spectroscopy and dynamics of the S_1_(nπ*) state

**Figure 2:**
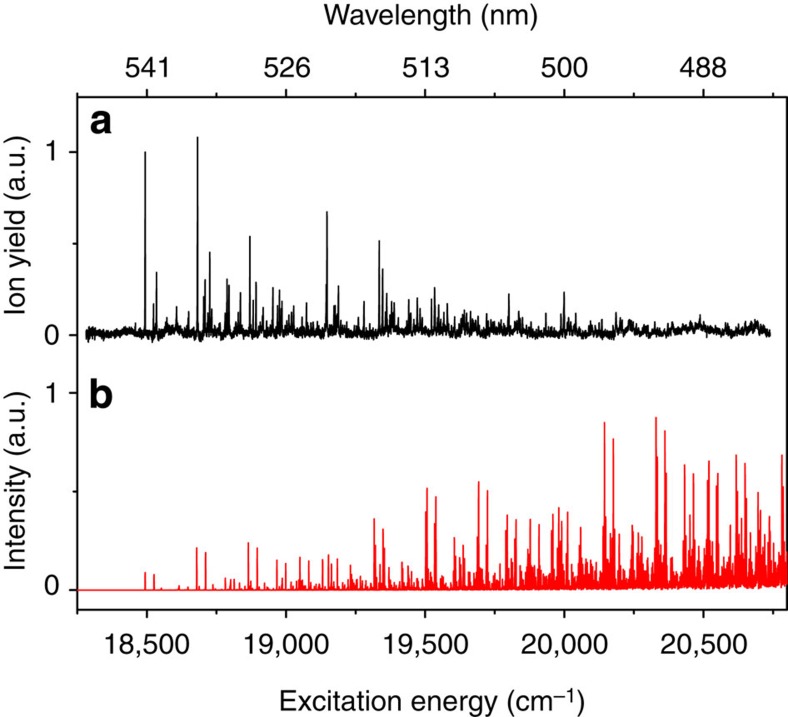
Excitation spectrum of S_1_(nπ*)←S_0_ transition of *tran*s-azobenzene. (**a**) Experimental jet-cooled excitation spectrum obtained using two-colour resonance enhanced two-photon ionization spectroscopy. The horizontal axis labels the frequency of the laser used for excitation of the molecule to the S_1_(nπ*) state, which is subsequently ionized by absorption of a 193-nm photon. (**b**) Excitation spectrum predicted within the Herzberg–Teller approximation of the electronic transition dipole moment using quantum chemical calculations of the equilibrium geometries, harmonic force fields of the ground and S_1_(nπ*) excited state, as well as electronic transition dipole derivatives. The spectrum has been generated by convolution of calculated stick spectra with 0.4-cm^−1^ bands, as found experimentally for the initial part of the excitation spectrum (see text).

Figure 2a shows the S_1_(nπ*)←S_0_ excitation spectrum of *trans*-azobenzene in the 18,250–20,750 cm^−1^ region, as detected by (1+1′) resonance enhanced two-photon ionization (RE2PI) spectroscopy. Compared with the best-resolved absorption spectrum reported so far, which was obtained in mixed crystals^34^, the resolution of the present spectrum is several orders of magnitude better. It thereby allows for the first time a detailed analysis of the changes in molecular structure upon excitation and their implications on the photochemical activity of the molecule. Experiments and quantum chemical calculations indicate that the molecule retains C_2h_ symmetry in both the ground and S_1_(nπ*) state. This implies that the S_1_(nπ*) (1^1^B_g_)←S_0_ (1^1^A_g_) transition is formally dipole-forbidden. The spectrum observed in Fig. 2a must therefore be built upon false origins involving vibrational modes of either a_u_ or b_u_ symmetry^34^ that couple the 1^1^B_g_ state to higher-lying excited singlet states of B_u_ or A_u_ symmetry. The first electronically excited states of B_u_ and A_u_ symmetry are S_2_ and S_5_, respectively, with calculated oscillator strengths of 0.616 and 0.008 (ref. 24). On the basis of this large difference in oscillator strengths, it is reasonable to assume that the false origins in the S_1_(nπ*)←S_0_ excitation spectrum involve a_u_ vibrations, as predicted previously^35^ and confirmed by our experimental data (*vide infra*).

Analysis of the excitation spectrum leads to the conclusion that it consists of several false origins upon which vibrational progressions involving totally symmetric a_g_ modes are built (see Supplementary Table 1), in particular ν_23_ (188 cm^−1^), ν_22_ (294 cm^−1^), ν_20_ (653 cm^−1^) and ν_19_ (853 cm^−1^). Importantly, we observe that ν_23_, which contains a significant amount of the NNC bend character, is the most active totally symmetric mode. This mode is directly associated with in-plane inversion of the two nitrogen atoms. Its motion thus relates directly to one of the suggested photoisomerization mechanisms, albeit that the actual photoisomerization process would involve a non-totally symmetric inversion mode. Nevertheless, the dominant activity of ν_23_ is significant, as it indicates that the hybridization and the related angles of the nitrogen atoms change considerably upon excitation. The importance of geometry changes along the inversion coordinate is also reflected in the activity of ν_20_, the second-most active mode. This mode involves predominantly CCC bending motions of the phenyl rings, but for a large part also NNC-bending motions.

Figure 2b displays the S_1_(nπ*)←S_0_ excitation spectrum predicted within the Herzberg–Teller approximation^36^. If we consider for the moment only the vibrational progressions involving totally symmetric a_g_ modes, qualitatively good agreement is observed between experimental and predicted intensities (see also Supplementary Table 4), although quantitatively the differences are larger than what one would have expected on the basis of studies of similar complexity^37^,^38^. In general, such differences indicate that the geometry changes upon excitation are not predicted accurately enough. To get an idea of the geometry changes that would lead to consistency with the experimentally observed excitation spectrum, we have employed the observed intensities of 
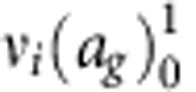
 transitions to ‘reconstruct’ the equilibrium geometry of the S_1_(nπ*) state^39^,^40^. In our experiments, we primarily observe Franck–Condon activity of bending vibrations and not of CC and CN stretch vibrations, which, as will be argued below, is attributed to the opening up of an additional non-radiative decay channel at these vibrational energies. As a result, the reconstructed geometry primarily reflects changes in the bond angles and not in bond lengths. Table 1 reports the most relevant geometrical parameters of ground and excited states as originally predicted by our calculations—which are in excellent agreement with previous calculations^21^,^26^—and as reconstructed from the excitation spectrum. This table indicates that the change in the NNC bond angle is considerably smaller than what is predicted by the calculations.

Above we have argued that the false origins are in principle associated with a_u_ or b_u_ vibrations. Consideration of the frequency intervals between the false origins and comparison with calculated vibrational frequencies rapidly leads to two conclusions. First, we find that these frequency intervals are in excellent agreement with what one would expect for the manifold of a_u_ vibrations, but cannot be reconciled with the frequencies of b_u_ vibrations (see Supplementary Table 3). Further support for an assignment to a_u_ vibrations comes from simulations of the rotational contours of the bands (*vide infra*). These contours can be reproduced very well under the assumption of a B_u_ (B_g_⊗a_u_)←A_g_ vibronic transition, but completely fail for an A_u_ (B_g_⊗b_u_)←A_g_ vibronic transition. We conclude that the S_1_(nπ*)←S_0_ transition obtains its intensity by vibronic coupling with the 1^1^B_u_ state, in agreement with *a priori* expectations. Second, it has to be concluded that the strong band at 18,493.8 cm^−1^ is the lowest-energy false origin and is associated with ν_44_, the lowest-frequency a_u_ vibration. The true S_1_(nπ*)←S_0_ 0–0 origin transition is not observed, but we can obtain a reasonably accurate estimate of its value by two different approaches, which both lead to the conclusion that it occurs at 18,471.7 cm^−1^ (see Supplementary Notes 1 and 2).

The S_1_(nπ*)←S_0_ excitation spectrum does not show only the 
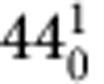
 false origin but also false origins associated with the 
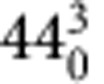
 and 
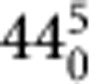
 transitions. These transitions are forbidden within the harmonic oscillator approximation, and can only obtain intensity by anharmonicity or Duschinsky mixing, that is, normal-mode rotation upon excitation. As the energy differences between the bands indicate that the anharmonicity is rather small, one has to conclude that Duschinsky mixing is the principal reason for the appearance of the overtone false origins. Quantum chemical calculations confirm this conclusion: they show that ν_44_ and ν_43_ become extensively mixed upon excitation.

Apart from ν_44_, several other a_u_ modes serve as false origins as well, most notably ν_43_, ν_42,_ ν_41_, ν_40_ and ν_37_ whose transitions are displaced from the 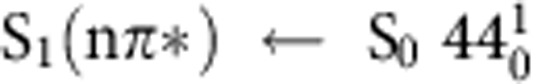 false origin by 29.9, 210.3, 388.3, 459.0 and 786.4 cm^−1^, respectively. Comparison of the relative intensities of the false origins (see Supplementary Table 5) shows that ν_44_ is by far the most effective in inducing intensity in the S_1_(nπ*)←S_0_ transition. It is well known that vibronic coupling leads to changes in the frequency of the coupling mode^41^,^42^. Compared with the uncoupled case, the frequency of the coupling mode is reduced in the lower state and increased in the upper state. Theory thus makes one expect that the relative frequency difference in the two states is a good measure for the vibronic coupling strength. Indeed, we find that the frequency of ν_44_ in S_1_ and S_2_ differs by more than a factor of two, while the other modes show much more moderate differences (see Supplementary Table 3). The conclusion that the S_1_(nπ*)←S_0_ transition is to a dominant extent enabled by ν_44_ is an important one. This mode is essentially a pure CNNC torsional mode, the mode along which isomerization has been proposed to occur. The overall picture that thus emerges from the present isolated molecule spectrum is one in which the S_1_(nπ*) state can only be accessed if simultaneously an a_u_ vibrational mode is excited, and that the most effective way to do so is to impart energy into the mode that involves torsion around the N=N double bond. It should be kept in mind that the present experiments have been performed under conditions in which vibrational levels in the ground state are not populated. At room temperature, however, low-frequency vibrations will be excited as well. Under such conditions, transitions can also occur in which the a_u_ vibrational quantum number is decreased^35^.

**Figure 3:**
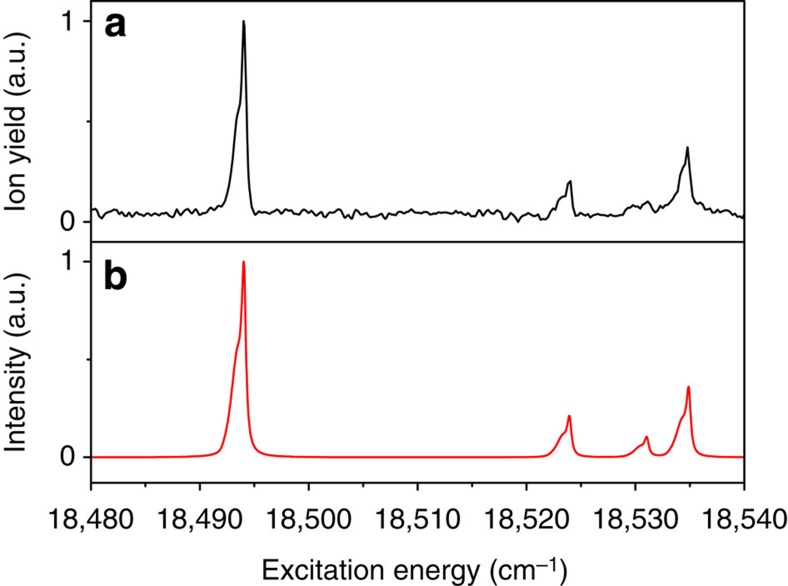
Rotational contour of bands in the S_1_(nπ*)←S_0_ excitation spectrum near the forbidden 0–0 transition. (**a**) Experimentally observed contours. (**b**) Simulated contours assuming a vibronic B_u_←A_g_ transition, a rotational temperature of 9 K and a homogeneous linewidth for individual rotational transitions of 0.4 cm^−1^.

Figure 3 shows fits of the rotational contour of bands in the initial part of the excitation spectrum that start from the vibrationless ground state and use rotational constants and electronic transition moments as calculated. Excellent agreement between the experiment and simulation is observed, leading to the conclusion that under our experimental conditions the rotational population distribution can be described by a rotational temperature of 9 K. More importantly, these simulations show that the homogeneous linewidth of the individual rotational transitions is 0.4 cm^−1^ corresponding to a lifetime of the lower rovibronic levels of S_1_ of about 13 ps (see Supplementary Note 3 for a further discussion of the fits). This lifetime is almost an order of magnitude longer than the lifetime reported so far in solution-phase experiments on the S_1_(nπ*) state^11^,^12^,^14^,^16^. The large difference between gas- and solution-phase experiments is remarkable, but, as argued below, is completely in line with the different experimental conditions under which the two types of experiments have been performed.

**Figure 4:**
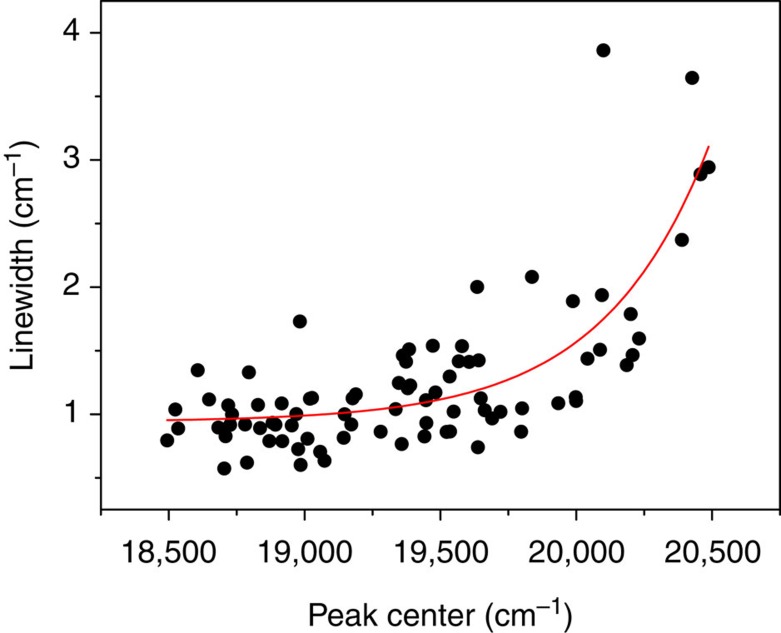
Influence of excitation energy on linewidths. Dependence of linewidths of bands in the S_1_(nπ*)←S_0_ excitation spectrum on excitation energy, the drawn line serving as a guide to the eye. For low excitation energies, bands have rather similar linewidths, but these start to increase markedly above ~19,250–19,500 cm^−1^. This increase gives evidence for an additional non-radiative decay channel that opens up for excess energies of ~2 kcal mol^−1^.

Figure 4 shows the dependence of the linewidths on the excitation energy, as determined from fits of the rotational contours. Although there is considerable scatter among the linewidths, it is clear that for low excitation energies the bands have a similar linewidth. However, for excitation energies above ~19,250–19,500 cm^−1^ the linewidths become larger, increasing to values of 4 cm^−1^ and larger around 20,500 cm^−1^. These observations strongly suggest that the decay dynamics of the S_1_(nπ*) state involve a non-radiative decay channel that opens up at about 750–1,000 cm^−1^ above the vibrationless level of the S_1_(nπ*) state. The same conclusion is reached when the experimentally observed vibronic intensities (Fig. 2a) are compared with theoretically predicted intensities (Fig. 2b). Up to ~19,250–19,500 cm^−1^ experiment and theory match well, but for higher excitation energies the experimental spectrum shows a progressive decrease of vibronic activity while theory predicts in contrast an increase in activity. Franck–Condon considerations for the ionization step of our experiments exclude any non-radiative decay mechanism that leaves azobenzene in an electronically excited state. Our experiments therefore show that the marked loss in intensity in the experimental spectrum is due to a very fast decay to the ground state.

The presence of a decay channel that only comes into play above a certain threshold energy explains the apparent mismatch between the lifetime measured in the present experiments and in solution. Time-resolved transient absorption and fluorescence experiments in solution differ in two aspects from the present experiments. First and most importantly, in solution molecules are excited at or near the absorption maximum, leading to excitation energies that are at least some 2,000 cm^−1^ above the 0–0 transition determined here. Second, the present experiments have been performed at an effective rotational temperature of 9 K—the vibrational temperature admittedly being somewhat higher—while solution-phase experiments have been performed at room temperature. Both conditions lead to a situation in which the solution-phase experiments’ vibronic levels are excited in a region well above the barrier for accessing the non-radiative decay channel discussed above. On a timescale of a few ps the effects of vibrational cooling are still limited^43^, and one would therefore expect that in the solution-phase experiments decay times are observed that are near the values estimated here for excitation of high-lying vibronic levels. This expectation is borne out by our experiments: the reported range of decay times of 0.6–2.6 ps corresponds to linewidths of 2–9 cm^−1^.

The photoisomerization mechanism of azobenzene after excitation of the S_1_(nπ*) state is still a subject of extensive debate. In the present study, we do not have direct access to the reactive part of the potential energy surface, but we do notice that our observations agree remarkably well with the relevant features of the potential energy surface as emerging from high-level *ab initio* calculations^24^,^26^. This pertains in particular to the presence of a ~750–1,000-cm^−1^ (2.1–2.9 kcal mol^−1^) barrier for accessing an additional non-radiative decay channel. Calculations find on the potential energy surface of the S_1_(nπ*) state a transition state with an activation energy of about 2 kcal mol^−1^. Conical intersections of S_1_ and S_0_ along the torsional pathway are found at more or less the same energy, while conical intersections along the inversion pathway occur at much higher energies^22^,^24^. We thus conclude that the experimentally observed barrier is in excellent agreement with the predicted activation energy, while the experimental conclusion that above the barrier the excited state decays ultrafast to the electronic ground state is entirely consistent with an S_0_/S_1_ conical intersection at similar energies as the activation energy. Taking into account that both experiment and theory agree that excitation is accompanied by a significant change of the NNC bond angle, it would appear that for the isolated molecule photoisomerization predominantly takes place via an inversion-assisted torsional pathway^3^ in which large changes in the CNNC dihedral angle occur simultaneously with smaller, but significant changes in the NNC angles. Importantly, changes along the CNNC torsional coordinate are intrinsically already promoted by the fact that excitation of the S_1_(nπ*) state occurs predominantly by simultaneously exciting the N=N double-bond torsional vibration.

### Spectroscopy and dynamics of the S_2_(ππ*) state

**Figure 5:**
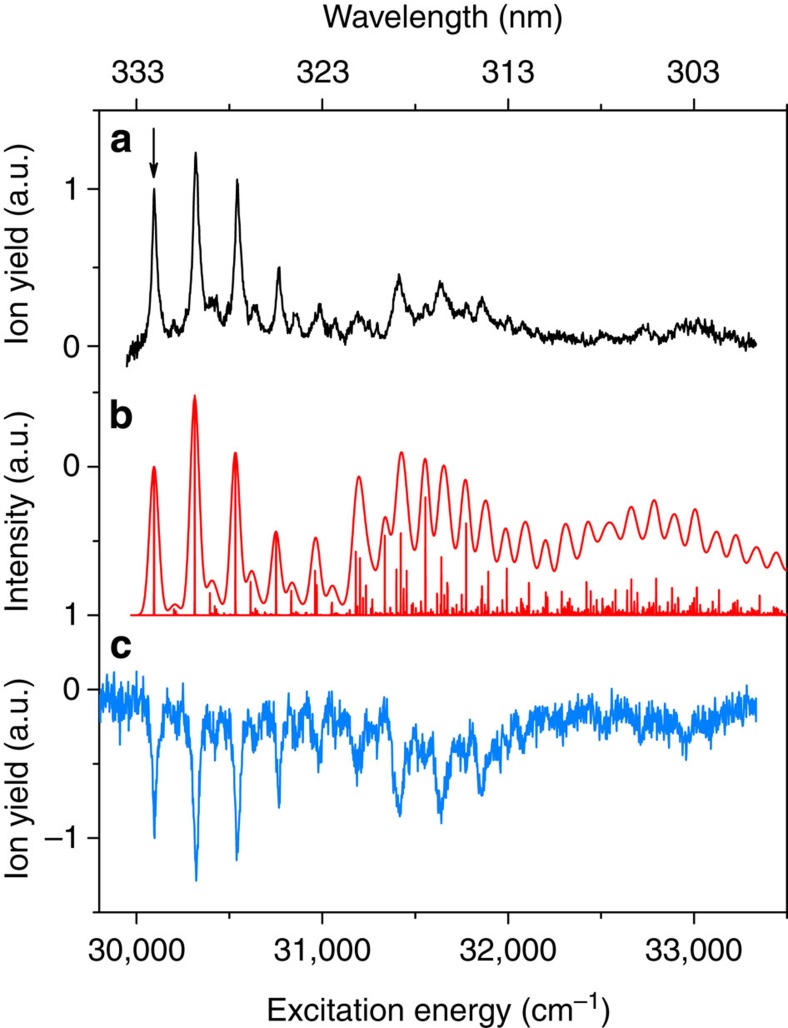
Excitation spectrum of S_2_(ππ*)←S_0_ transition of *trans*-azobenzene. (**a**) Experimental jet-cooled excitation spectrum obtained using two-colour resonance enhanced two-photon ionization spectroscopy. The horizontal axis labels the frequency of the laser used for excitation of the molecule to the S_2_(ππ*) state, which is subsequently ionized by absorption of a 193-nm photon. (**b**) Excitation spectrum predicted within the Franck–Condon approximation using quantum chemical calculations of the equilibrium geometries and harmonic force fields of the ground and S_2_(ππ*) excited states. The spectrum has been generated by convolution of calculated stick spectra with 32-cm^−1^ bands, as found experimentally for the initial part of the excitation spectrum (see text). (**c**) Ultraviolet–ultraviolet depletion spectrum of the S_2_(ππ*) state using the transition at 30,096.6 cm^−1^ indicated by the arrow as a probe.

The excitation spectrum of jet-cooled *trans*-azobenzene to the second excited state is shown in Fig. 5a. We find that the 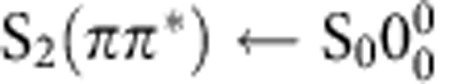 transition is located at 30,096.6±0.2 cm^−1^. Qualitatively, the spectrum is similar to previously reported spectra of azobenzene in dibenzyl crystals^44^ and in the vapour phase^45^,^46^, but the resolution is significantly higher. This resolution in fact enables us to conclude that the widths of the bands are lifetime limited. Fits of the origin band with a Lorentzian find a width of 32.0±1.0 cm^−1^ corresponding to a lifetime of 166±5 fs, which is in excellent agreement with the lifetime reported in femtosecond time-resolved photoelectron spectroscopic studies (170 fs)^9^, and time-resolved fluorescence studies (110 fs)^47^.

Figure 5a shows that the most active mode in the S_2_(ππ*)←S_0_ spectrum is ν_23_ (223 cm^−1^), similar to what is observed for excitation of the S_1_(nπ*) state. In agreement with the previous low-resolution studies^44^, we also see a prominent role of ν_12_ (1,315 cm^−1^). However, at the same time the present resolution enables us to observe the activity of many other modes that so far have not been reported, and thereby to determine in greater detail the structural changes occurring upon excitation to this state. Detailed assignment of the bands in the excitation spectrum (see Supplementary Table 2) shows that they nearly all involve fundamentals of totally symmetric vibrations and their combinations. The only exception is the low-intensity band at 103 cm^−1^ from the origin, which also appears in combination with other bands. This band must be attributed to the 
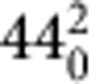
 transition. The activity of this mode indicates that it changes considerably upon excitation, as was also concluded for the S_1_(nπ*) state.

Comparison of the experimental spectrum with the theoretically predicted spectrum (Fig. 5b) shows very good agreement, and indicates that the broadening of the experimental spectrum at higher energy is due to spectral congestion. In contrast to simulations of the S_1_(nπ*)←S_0_ spectrum, the predicted intensities now follow quite closely the experimentally observed intensities. Accordingly, we find that reconstruction of the equilibrium geometry of the S_2_(ππ*) state from the experimentally observed intensities leads to minor changes in bond lengths and bond angles (Table 1).

The relaxation pathways and dynamics of the S_2_(ππ*) state have been the subject of a large amount of experimental and theoretical studies. In several of these studies, it has been suggested that other electronically excited states are involved in the decay of the S_2_(ππ*) state^9^,^24^,^26^. Generally, different electronic states have different ionization cross-sections. One might thus see signatures of an excitation-energy-dependent involvement of other electronically excited states by comparing the (1+1′) RE2PI excitation spectrum—for which intensities depend on both excitation and ionization cross-section—with the absorption spectrum of the S_2_(ππ*) state obtained by ultraviolet–ultraviolet depletion spectroscopy for which intensities only depend on the excitation cross-section. However, comparison of the ultraviolet–ultraviolet depletion spectrum shown in Fig. 5c with the RE2PI spectrum depicted in Fig. 5a does not give any indication of significant differences between the two spectra. This similarity also precludes significant contributions from decay channels leading to states that cannot be ionized, as would occur upon internal conversion to the ground state. We therefore conclude that our observations are consistent with an efficient ultrafast S_1_←S_2_ internal conversion, but cannot make definite statements on further details of this pathway.

## Discussion

High-resolution nanosecond laser spectroscopy has enabled us to scan the potential energy surfaces of electronically excited states of *trans*-azobenzene. These studies have unveiled key aspects of how the structure of the molecule is changed upon photoexcitation, and how photoisomerization takes place. From these studies a picture has been established in which (a) excitation of the S_1_(nπ*) state is primarily accompanied by in-plane changes in the hybridization angles around the nitrogen atoms, (b) excitation of this dipole-forbidden state occurs most effectively if it involves the CNNC torsional mode, the mode that is directly involved in the torsional isomerization pathway and (c) photoisomerization in the S_1_(nπ*) state occurs via an inversion-assisted torsional pathway with a barrier of ~2 kcal mol^−1^ separating the local minimum of the S_1_(nπ*) state from a conical intersection with the ground state at similar energies as the barrier. Similarly, we have determined the structural changes occurring upon excitation to the strongly absorbing S_2_(ππ*) state. Comparison of the absorption and excitation spectra of this state have led to the conclusion that, at least from these spectra, no indication can be found for the importance of dynamical processes other than internal conversion to the S_1_(nπ*) state.

Elucidation of the details of the potential energy surfaces of the S_1_(nπ*) and S_2_(ππ*) states and their associated dynamics is important, since they ultimately determine the operation of azobenzene in photofunctional devices. In this respect, the present results could form the basis for a further rational design of novel molecular devices that use this important building block. At a more general level, the present studies have shown the important benefits high-resolution spectroscopy can offer for the study of (ultra)fast photodynamics. They should provide further impetus for similar studies on the vast amount of photoresponsive systems that so far have not been considered, because their lifetimes were considered to be prohibitively short.

## Methods

### Experimental details

In our studies, excitation spectra of cold isolated molecules were recorded by two-colour (1+1′) RE2PI spectroscopy on *trans*-azobenzene seeded in a supersonic expansion of neon. For this purpose, *trans*-azobenzene (Sigma-Aldrich) was placed in an oven that was heated up to 145 °C and expanded into vacuum with 2 bars of Ne via a pulsed nozzle with a 0.5-mm orifice (General Valve Iota One system). Following the supersonic free jet expansion, a 2-mm conical skimmer skimmed the expansion prior to entering the ionization chamber. Ions were detected using a reflectron time-of-flight mass spectrometer (R.M. Jordan Co.).

In the two-colour RE2PI experiments, a frequency-doubled dye laser (Sirah Precision Scan) pumped by a Nd:YAG laser (Spectra Physics Lab 190) operating at 30 Hz was used for excitation. Typical pulse energies were 2–3 mJ, but when necessary these were attenuated to avoid saturation. Ionization occurred by means of an ArF excimer laser (Neweks PSX-501) that typically produces pulses with energies of 5 mJ per pulse. The two lasers are introduced into the spectrometer in a counter-propagating fashion without further focusing. The pulsed nozzle and the two laser systems were synchronized by a delay generator (Stanford Research Systems DG535). For the ultraviolet–ultraviolet depletion experiments, another laser system consisting of a frequency-doubled dye laser (Sirah Cobra-Stretch) pumped by a second Nd:YAG laser (Spectra Physics Lab 190) was employed. In these experiments, the depletion laser, which is scanned over the excitation energy of interest, was fired 100–200 ns prior to the lasers creating the (1+1′) RE2PI probe signal at a fixed excitation wavelength.

### Computational details

Geometry optimization followed by calculation of the harmonic force field was performed for the S_0_ ground state and the S_1_(nπ*) and S_2_(ππ*) electronically excited states using time-dependent density functional theory. These calculations were performed at the B3LYP/6-31G(d,p) level utilizing the Gaussian09 suite of programs^48^. The same program was employed for simulation of the vibrationally resolved excitation spectra within the Herzberg–Teller approximation. For comparison with experimental spectra, a scaling factor of 0.961 was used for the predicted frequencies.

## Author contributions

E.M.M.T. designed and performed the experiments, performed the calculations and analysed the data. S.A. assisted with the experiments, calculations and their interpretation. S.S. and A.V. designed and performed initial experiments on the S_2_(ππ*) state that formed the basis for the experiments reported here. F.Z. helped with the interpretation of the experimental data. W.J.B. designed the experiments and supervised the experiments, calculations and data analysis. E.M.M.T. and W.J.B. co-wrote the manuscript with input from S.A. and F.Z.

## Additional information

**How to cite this article:** Tan, E. M. M. *et al.* Fast photodynamics of azobenzene probed by scanning excited-state potential energy surfaces using slow spectroscopy. *Nat. Commun.* 5:5860 doi: 10.1038/ncomms6860 (2014).

## Supplementary Material

Supplementary Information Supplementary Figures 1-2, Supplementary Tables 1-5, Supplementary Notes 1-3 and Supplementary References

## Figures and Tables

**Figure 1:**
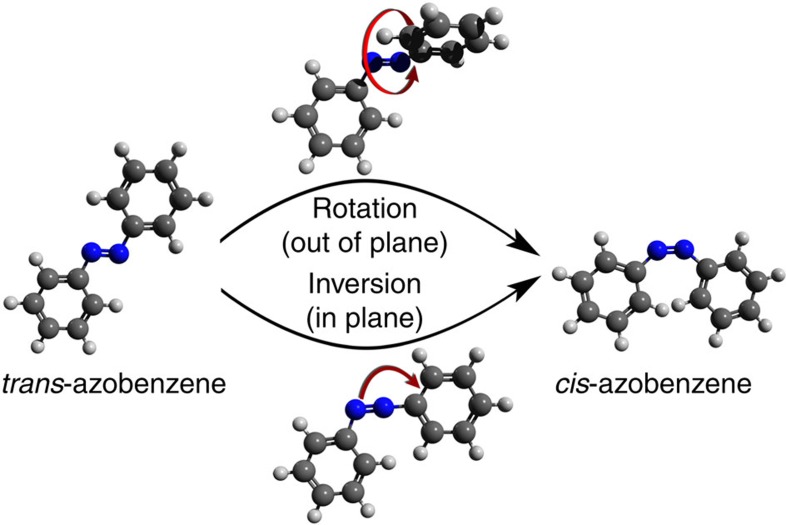
Isomerization pathways of *trans*-azobenzene. Traditionally, isomerization of *trans*-azobenzene has been envisaged to occur via either a rotation or inversion mechanism. In the first mechanism torsion around the N=N bond occurs; in the second mechanism the phenyl rings move in the plane of the molecule towards each other.

**Table 1:** Relevant geometrical parameters of *trans*-azobenzene in the S_0_, S_1_(nπ*) and S_2_(ππ*) states as determined from the experiment and predicted by quantum chemical calculations.

	**S**_**0**_ experiment*	**S**_**1**_ **theory**†	**S**_**1**_ **reconstructed**†^,^‡	**S**_**2**_ **theory**†	**S**_**2**_ **reconstructed**§
*d*(C–N) (Å)	1.428	1.358	1.365	1.369	1.373
*d*(N=N) (Å)	1.260	1.251	1.262	1.351	1.324
∠N=N–C (°)	113.7	130.5	124.3	111.5	112.2
∠C–C–N (°)	124.8	122.2	123.3	124.4	124.6

^*^Structural parameters taken from gas-phase electron diffraction^49^.

^†^Present work.

^‡^Determined from experimental intensities of 
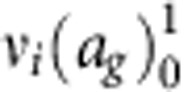
 transitions for modes *i*=23–19, and the intensities predicted by the calculations for modes *i*=18–6 (see Supplementary Table 4).

^§^Determined from experimental intensities of 
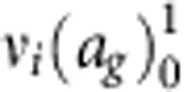
 transitions for modes that have been observed and identified in the S_2_(ππ*)←S_0_ spectrum. Modes that have not been observed have been given zero intensity (see Supplementary Table 4).
