# Supplementary material for: Fast photodynamics of azobenzene probed by scanning excited-state potential energy surfaces using slow spectroscopy
Source: Nat Commun. 2015 Jan 6;6:5860. doi: 10.1038/ncomms6860 (PMC4308720; doi:10.1038/ncomms6860)
Supplement: Supplementary Information — Supplementary Figures 1-2, Supplementary Tables 1-5, Supplementary Notes 1-3 and Supplementary References [file ncomms6860-s1.pdf]

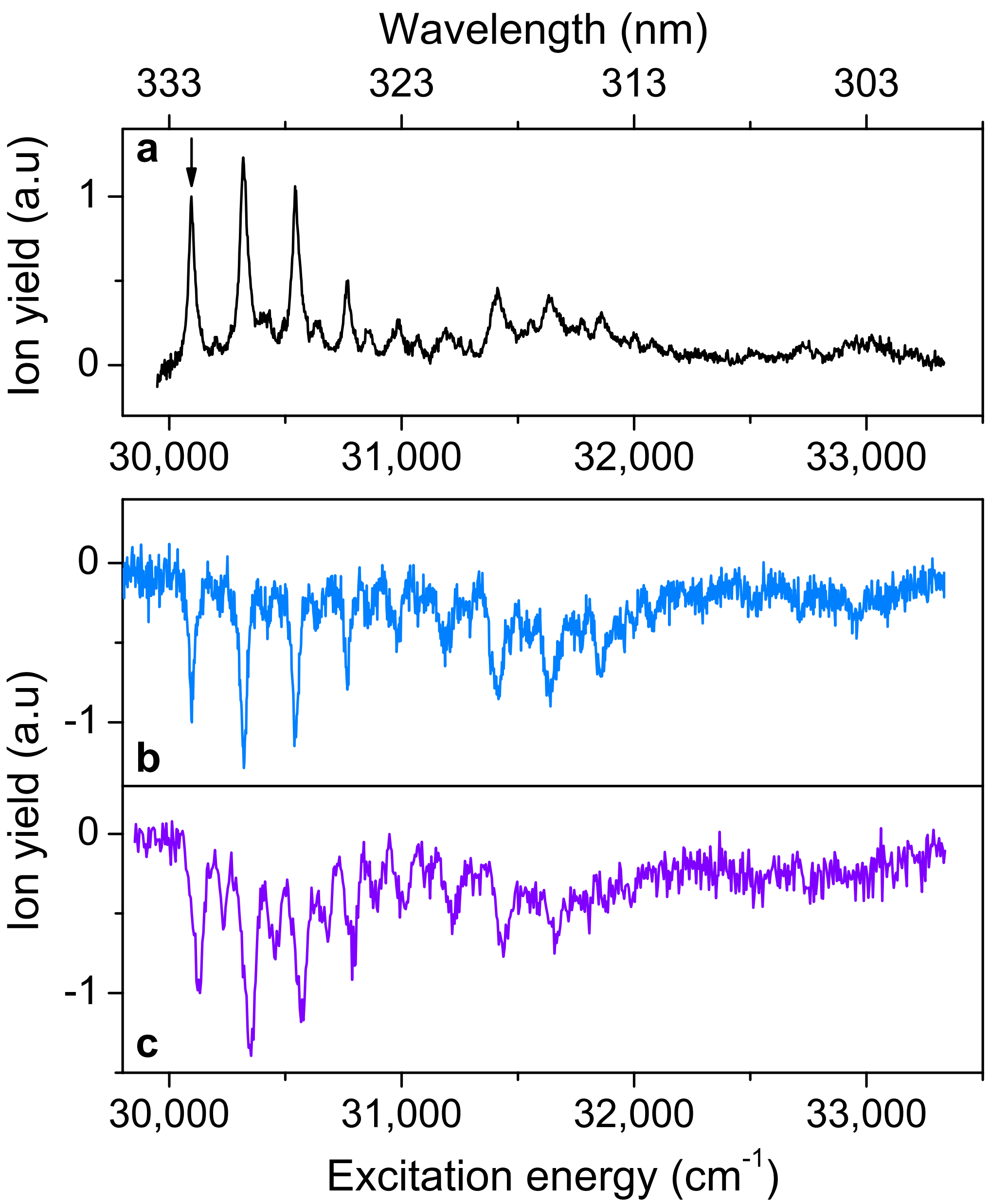

Supplementary Figure 1: UV-UV depletion experiments. (a) $S_2(\pi\pi^*) \leftarrow S_0$ (1+1') RE2PI excitation spectrum. The horizontal axis labels the frequency of the laser used for excitation of the molecule to the $S_2(\pi\pi^*)$ state which is subsequently ionized by absorption of a 193 nm photon. (b) UV-UV depletion spectrum of the $S_2(\pi\pi^*)$ state using the transition at 30096.6 cm^{-1} indicated by the arrow as a probe. (c) UV-UV depletion spectrum of the $S_2(\pi\pi^*)$ state using the transition at 18458.8 cm^{-1} observed in the $S_1(n\pi^*) \leftarrow S_0$ (1+1') RE2PI excitation spectrum as a probe.

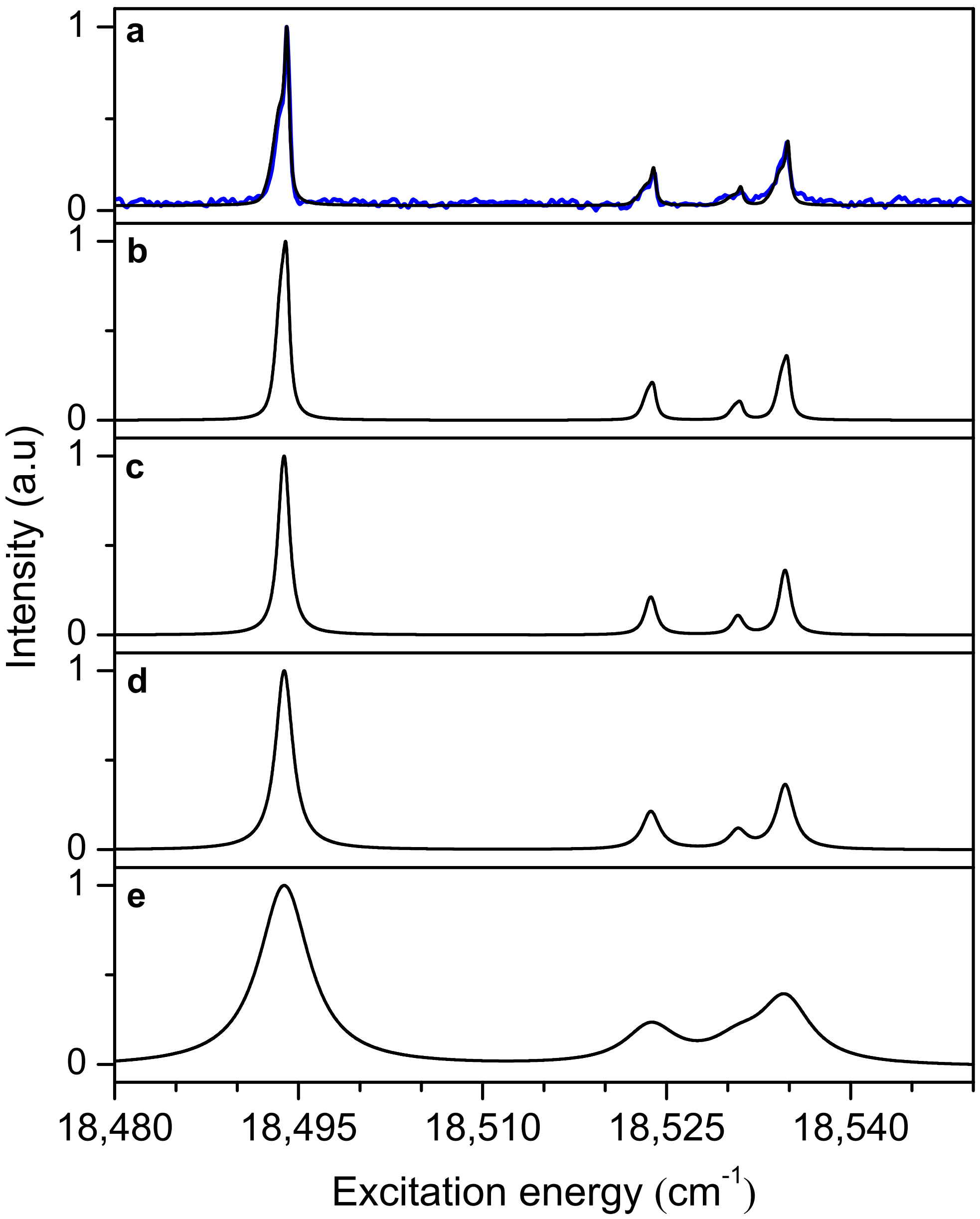

Supplementary Figure 2: Rotational contour simulations. (a) Experimental spectrum of the first four bands in the $S_1(n\pi^*) \leftarrow S_0$ (1+1') RE2PI excitation spectrum (blue) superimposed on a simulated spectrum using a temperature of 9K and a Lorentzian line width for rotational transitions of 0.4 cm^{-1}. Spectra (b)-(e) show spectra simulated with various other line widths (with in parentheses associated lifetimes) at temperatures optimized as to give as good an agreement as possible with the experimental spectrum. (b) 0.6 cm^{-1} (8 ps); 4K (c) 1.0 cm^{-1} (5 ps); 1K (d) 1.7 cm^{-1} (3 ps); 0K (e) 5.0 cm^{-1} (1 ps), 0K.

Supplementary Table 1. Assignment of bands observed in the $S_1(n\pi^*) \leftarrow S_0$ (1+1') RE2PI excitation spectrum of *trans*-azobenzene. Bands are reported with respect to 18471.7 cm^{-1}, the excitation energy of the forbidden 0-0 transition as determined from the 44_0^1, 44_0^3, and 44_0^5 bands. Normal modes are labelled following the numbering employed in Refs. 1 and 2.

Excitation energy (cm^{-1})	Assignment	Intensity (%)
-35.0	44_1^0	7
0.0	0_0^0	0
22.1	44_0^1	100
52.0	43_0^1	17
59.1	$43_0^1 44_1^1$	6
63.0	44_0^3	34
96.5	$43_0^1 44_0^2$	7
100.4	44_0^5	10
134.8	$43_0^2 44_0^1$	15
177.1	$44_0^3 66_0^2$	9
178.7	$43_0^2 44_0^3$	13
210.4	$44_0^1 23_0^1$	108
232.4	42_0^1	21
237.5	$43_0^1 23_0^1$	30
247.2	$43_0^1 44_1^1 23_0^1$	12
254.3	$44_0^3 23_0^1$	45
261.2	$43_0^1 44_0^4 66_0^2$	14
309.4	$42_0^1 43_0^1 44_0^1$	12
316.2	$44_0^1 22_0^1$	31
323.1	$43_0^2 44_0^1 23_0^1$	27
349.0	$43_0^1 22_0^1$	7
356.6	$44_0^3 22_0^1$	12
364.3	$43_0^2 44_0^3 23_0^1$	23
398.5	$44_0^1 23_0^2$	54
410.4	41_0^1	19
420.6	$42_0^1 23_0^1$	29
446.0	$44_0^3 23_0^2$	15
459.6	$42_0^1 44_0^2 23_0^1$	8
481.1	40_0^1	26
497.7	$42_0^1 43_0^1 44_0^1 23_0^1$	16
504.5	$44_0^1 22_0^1 23_0^1$	25
511.9	$43_0^2 44_0^1 23_0^2$	13
513.8	$44_0^1 66_0^2 23_0^2$	19
539.7	$43_0^1 22_0^1 23_0^1$	11

547.9	$44^3_0 22^1_0 23^1_0$	12
555.6	$43^2_0 44^3_0 23^2_0$	16
585.8	$44^1_0 23^3_0$	10
601.6	$41^1_0 23^1_0$	18
672.6	$40^1_0 23^1_0$	28
675.5	$44^1_0 20^1_0$	67
700.4	$41^1_0 22^1_0$	16
704.8	$43^1_0 20^1_0$	16
711.9	$42^1_0 22^1_0 23^1_0$	11
716.5	$44^3_0 20^1_0$	27
774.8	$40^1_0 22^1_0$	5
788.3	$41^1_0 23^2_0$	10
808.5	37^1_0	18
829.8	$44^3_0 43^2_0 20^1_0$	6
863.2	$44^1_0 20^1_0 23^1_0$	52
875.3	$44^1_0 19^1_0$	36
885.5	$42^1_0 20^1_0$	13
890.1	$43^1_0 20^1_0 23^1_0$	23
907.7	$44^3_0 20^1_0 23^1_0$	18
917.0	$44^3_0 19^1_0$	18
969.2	$44^1_0 20^1_0 22^1_0$	19
975.2	$43^2_0 44^1_0 20^1_0 23^2_0$	12
977.4	$44^1_0 66^2_0 20^1_0 23^2_0$	12
999.8	$37^1_0 23^1_0$	20
1010.0	$44^3_0 20^1_0 22^1_0$	15
1018.4	$44^3_0 43^2_0 20^1_0 23^1_0$	10
1051.1	$44^1_0 20^1_0 23^2_0$	20
1062.3	$44^1_0 19^1_0 23^1_0$	26
1076.7	$43^1_0 20^1_0 23^2_0$	17
1095.4	$44^3_0 20^1_0 23^2_0$	12
1107.9	$44^3_0 19^1_0 23^1_0$	17
1134.1	$40^1_0 20^1_0$	10
1169.9	$44^1_0 19^1_0 22^1_0$	14
1191.3	$37^1_0 23^2_0$	13
1250.0	$44^1_0 19^1_0 23^2_0$	12
1328.9	$44^1_0 20^2_0$	22
1370.0	$44^3_0 20^2_0$	9
1462.1	$37^1_0 20^1_0$	12
1516.2	$44^1_0 20^2_0 23^1_0$	12
1527.6	$44^1_0 19^1_0 20^1_0$	23
1569.5	$44^3_0 19^1_0 20^1_0$	12
1714.0	$44^1_0 19^1_0 20^1_0 23^1_0$	12

Supplementary Table 2. Assignment of bands observed in the $S_2(\pi\pi^*) \leftarrow S_0$ (1+1') RE2PI excitation spectrum of *trans*-azobenzene. Bands are reported with respect to 30096.6 cm^{-1}, the excitation energy of the 0-0 transition.

Excitation energy (cm^{-1})	Assignment	Intensity (%)
0	0_0^0	100
103	44_0^2	18
223	23_0^1	122
308	22_0^1	15
333	$44_0^2 23_0^1$	23
447	23_0^2	106
534	$22_0^1 23_0^1$	29
668	23_0^3	51
756	$22_0^1 23_0^2$	23
866	19_0^1	13
892	23_0^4	25
971	$22_0^1 23_0^3$	18
1055	$19_0^1 23_0^1$	12
1092	15_0^1	19
1149	13_0^1	13
1200	$18_0^1 23_0^1$	14
1315	12_0^1	42
1372	$13_0^1 23_0^1$	29
1459	8_0^1	26
1538	$12_0^1 23_0^1$	42
1596	$13_0^1 23_0^2$	27
1639	6_0^1	20
1682	$8_0^1 23_0^1$	27
1761	$12_0^1 23_0^2$	31
1862	$6_0^1 23_0^1$	20
1906	$8_0^1 23_0^2$	21
1984	$12_0^1 23_0^3$	19
2180	$12_0^1 19_0^1$	13
2409	$12_0^1 15_0^1$	12
2631	12_0^2	15
2776	$8_0^1 12_0^1$	13
2855	$12_0^2 23_0^1$	19
3079	$12_0^2 23_0^2$	11

Supplementary Table 3. Theoretically predicted and (in parentheses) experimentally observed vibrational frequencies in the ground state of *trans*-azobenzene,[1,3] and in its $S_1(n\pi^*)$ and $S_2(\pi\pi^*)$ excited states.

a_g	S_0	S_1	S_2	Description
ν_{23}	215 (219)	185 (188)	219 (223)	NNC bend
ν_{22}	295	288 (294)	300 (308)	CCN bend
ν_{21}	602 (614)	600	567	CCC bend
ν_{20}	657 (670)	637 (653)	633	CCC bend + NNC bend
ν_{19}	903 (913)	827 (853)	866 (866)	CCC bend + NNC bend
ν_{18}	977 (1003)	962	955	CC stretch + CCC bend
ν_{17}	1005 (1023)	1001	983	CCH bend + CC stretch
ν_{16}	1058 (1071)	1066	1052	CCH bend
ν_{15}	1122 (1146)	1130	1108 (1092)	CCH bend + NC stretch
ν_{14}	1139 (1158)	1136	1124	CCH bend
ν_{13}	1165 (1184)	1171	1140 (1149)	CCH bend + NC stretch
ν_{12}	1291 (1315)	1286	1241 (1315)	CCH bend
ν_{11}	1316 (1379)	1310	1281	CCH bend + CC stretch
ν_{10}	1429 (1443)	1436	1328	CCH bend + NN stretch
ν_9	1456 (1473)	1447	1425	CCH bend + CC stretch
ν_8	1497 (1493)	1532	1436 (1459)	NN stretch + CCH bend + CC stretch
ν_7	1578	1572	1487	CC stretch + CCH bend + NN stretch
ν_6	1593 (1595)	1612	1579 (1639)	CC stretch + CCH bend

b_g	S_0	S_1	S_2	Description
ν_{33}	104	149	114	NC torsion
ν_{32}	250 (251)	388	263	CN oop bend
ν_{31}	406 (403)	415	388	ring oop def
ν_{30}	473	581	466	CN oop bend + CH oop bend
ν_{29}	672	667	633	CH oop bend
ν_{28}	751 (755)	739	745	CH oop bend
ν_{27}	831 (836)	794	794	CH oop bend
ν_{26}	913	851	879	CH oop bend
ν_{25}	951 (938)	925	939	CH oop bend
ν_{24}	969 (968)	943	954	CH oop bend

a_u	S_0	S_1	S_2	Description
ν_{44}	23 (12)	29 (22)	54 (51)[b]	CNNC torsion[a]
ν_{43}	59	61 (52)	63	CN torsion (rotation of phenyls)[a]
ν_{42}	296	223 (232)	258	NN torsion + CN oop bend + CH oop bend
ν_{41}	403	403 (410)	392	ring oop def
ν_{40}	539 (545)	469 (481)	507	NN torsion + CN oop bend + CH oop bend
ν_{39}	679 (689)	659	665	CH oop bend
ν_{38}	769 (776)	719	762	CH oop bend + CN oop bend
ν_{37}	831 (850)	788 (809)	812	CH oop bend
ν_{36}	917 (927)	843	880	CH oop bend
ν_{35}	951	925	943	CH oop bend
ν_{34}	969 (985)	943	958	CH oop bend

[a] Description based on normal mode in S_1. However, in S_0 and S_2 the description of ν_{44} and ν_{43} is switched, *i.e.*, in these states ν_{44} has CN torsion (rotation of phenyls) character while ν_{43} has CNNC torsional character.

[b] Taken as half of the two-quanta transition.

b_u	S_0	S_1	S_2	Description
ν_{66}	80	56 (56)	78	NNC bend + CCN bend
ν_{65}	510 (521)	452	504	CCN bend + NNC bend
ν_{64}	525 (545)	517	516	CCC ring bend + CCN bend
ν_{63}	607 (615)	604	578	CCC ring bend
ν_{62}	806 (813)	816	796	CCC ring bend + NC stretch
ν_{61}	976 (1000)	955	950	CCC ring bend
ν_{60}	1005 (1020)	1001	982	CCH bend
ν_{59}	1061 (1072)	1063	1059	CCH bend
ν_{58}	1133 (1152)	1136	1128	CCH bend
ν_{57}	1139 (1160)	1142	1133	CCH bend
ν_{56}	1212 (1223)	1267	1239	CCH bend + CC stretch + NC stretch
ν_{55}	1284 (1300)	1285	1292	CCH bend + CCN bend
ν_{54}	1313 (1399)	1310	1326	CCH bend + CC stretch
ν_{53}	1436 (1456)	1428	1413	CCH bend + CC stretch
ν_{52}	1468 (1486)	1449	1446	CCH bend + CN stretch + CC stretch
ν_{51}	1572 (1585)	1530	1480	CC stretch + CCH bend
ν_{50}	1589	1562	1573	CC stretch + CCH bend

Supplementary Table 4. Experimentally observed and theoretically predicted intensities of $\nu_i(a_g)_0^1$ transitions in S_1(nπ*) ← S_0 and S_2(ππ*) ← S_0 excitation spectra. Experimentally observed frequencies and intensities are given in parentheses. Intensities are given with respect to the intensity of the 44_0^1 false origin in case of S_1 and with respect to the 0_0^0 transition in case of S_2.

a_g	S_1	Intensity (%)	S_2	Intensity (%)
ν_{23}	185 (188)	238 (108)	219 (223)	144 (122)
ν_{22}	288 (294)	68 (31)	300 (308)	15 (15)
ν_{21}	600	2	567	0
ν_{20}	637 (653)	169 (67)	633	0
ν_{19}	827 (853)	250 (36)	866 (866)	29 (13)
ν_{18}	962	11	955	7
ν_{17}	1001	0	983	0
ν_{16}	1066	6	1052	4
ν_{15}	1130	34	1108 (1092)	37 (19)
ν_{14}	1136	1	1124	13
ν_{13}	1171	7	1140 (1149)	21 (13)
ν_{12}	1286	16	1241 (1315)	55 (42)
ν_{11}	1310	0	1281	1
ν_{10}	1436	11	1328	0
ν_9	1447	2	1425	0
ν_8	1532	2	1436 (1459)	9 (26)
ν_7	1572	8	1487	2
ν_6	1612	33	1579 (1639)	21 (20)

Supplementary Table 5. Experimentally observed and theoretically predicted intensities of $\nu_i(a_u)_0^1$ transitions in the $S_1(n\pi^*) \leftarrow S_0$ excitation spectrum. Experimentally observed frequencies and intensities are given in parentheses. Intensities are given with respect to the intensity of the 44_0^1 false origin.

a_u	S_1	Intensity (%)
ν_{44}	29 (22)	100 (100)
ν_{43}	61 (52)	89 (17)
ν_{42}	223 (232)	21 (21)
ν_{41}	403 (410)	21 (19)
ν_{40}	469 (481)	14 (26)
ν_{39}	659	1
ν_{38}	719	1
ν_{37}	788 (809)	17 (18)
ν_{36}	843	0
ν_{35}	925	1
ν_{34}	943	0

Supplementary Note 1: Determination of $S_1(n\pi^*) \leftarrow S_0$ 0-0 transition energy.

The frequency of the $S_1(n\pi^*) \leftarrow S_0$ 0_0^0 transition has been determined by two different approaches. The first approach is based on the observation of the 44_0^3 and 44_0^5 transitions at 18534.7 and 18572.1 cm^{-1}, respectively. Using the vibrational energy expression for an anharmonic oscillator, one finds that the v=1 level of ν_{44} is at 22.1 cm^{-1}, and thereby that the $S_1(n\pi^*) \leftarrow S_0$ 0_0^0 transition is at 18471.7 cm^{-1}. In a second approach we consider the very weak band at 18458.8 cm^{-1}. UV-UV depletion experiments[4] that use this transition as a probe and the strong $S_2(\pi\pi^*) \leftarrow S_0$ transition as a pump demonstrate unambiguously that the band is associated with the 44_1^0 hot

band transition (see Supplementary Note 2). As yet, the ground-state frequency of ν_{44} has not been determined experimentally, but high-level calculations give values that range from 26.1 (DFT),[5] 18 (CASSCF),[2] to 11.9 cm^{-1} (MP2).[5] Taking the MP2 value as the most accurate one, the $S_1(n\pi^*) \leftarrow S_0\ 0_0^0$ transition can then be placed at 18471 cm^{-1}. It is gratifying to note that both approaches lead to a very similar value, which moreover is remarkably close to the value of 18520 cm^{-1} extrapolated in ref. 6 on the basis of gas-phase spectra obtained at 152 °C.[7]

Supplementary Note 2: UV-UV depletion experiments on $S_1(n\pi^*) \leftarrow S_0$ hot band.

In the $S_1(n\pi^*) \leftarrow S_0$ (1+1') RE2PI excitation spectrum a very weak band is observed at 18458.8 cm^{-1}. In order to assign this band we have performed UV-UV depletion experiments in which we use this transition as the probe, *i.e.*, we create a constant ion signal using an excitation frequency of 18458.8 cm^{-1} with which we excite *trans*-azobenzene and 193 nm to ionize it. We fire an additional laser (designated as the pump) 100-200 ns before the lasers that create the probe signal. Scanning the pump laser then leads to a depletion of this signal as a result of the depopulation of the ground state each time the pump laser is in resonance with a transition of the selected species. It should be noticed that in such an experiment *trans*-azobenzene in its vibrationless ground state is another species than *trans*-azobenzene in a vibrationally-excited ground state. In Supplementary Figure 1a the $S_2(\pi\pi^*) \leftarrow S_0$ (1+1') RE2PI excitation spectrum is displayed. Using a probe excitation wavelength of 30096.6 cm^{-1} which is associated with the $S_2(\pi\pi^*)$ 0-0 transition, a UV-UV depletion spectrum is observed (Supplementary Figure 1b) that exactly mirrors the regular excitation spectrum, thus demonstrating that all transitions in the regular (1+1') RE2PI

excitation spectrum are associated with one single species. A different result is obtained when the 18458.8 cm^{-1} band is employed (Supplementary Figure 1c). Although the UV-UV depletion spectrum resembles the regular (1+1') RE2PI spectrum, all bands are shifted by 30.1 cm^{-1}. This proves that the 18458.8 cm^{-1} band should be assigned as a hot band. Consideration of vibrational frequencies in combination with the temperature of molecules in the supersonic beam rapidly lead to the conclusion that the transition must be assigned to the $S_1(n\pi^*) \leftarrow S_0\ 44_1^0$ hot band.

Supplementary Note 3: Rotational contour simulations.

In order to determine the lifetime of the lower vibronic levels simulations have been performed of rotational contours of transitions to these levels using PGOPHER.[8] Excellent agreement can be obtained between experimentally observed and theoretically predicted contours if it is assumed that the rotational population distribution can be described by a rotational temperature of 9K and that the homogeneous line width of the individual rotational transitions is 0.4 cm^{-1}. The latter value implies a lifetime of about 13 ps, which is considerably longer than lifetimes reported up till now. We have therefore also performed simulations in which we fix the line width and optimize the rotational temperature as to come as close as possible to the experimentally observed rotational contour (see Supplementary Figure 2). In these simulations we find that the agreement between experiment and simulation is considerably worse, and that we need to employ unrealistic values for the temperature. This pertains in particular for panels (d) and (e) that employ lifetimes reported in solution-phase experiments.

Supplementary References

1. Stepanić, V., Baranović, G. & Smrečki, V. Structure and vibrational spectra of conjugated acids of *trans*- and *cis*-azobenzene. *J. Mol. Struct.* **569,** 89–109 (2001).
2. Gagliardi, L., Orlandi, G., Bernardi, F., Cembran, A. & Garavelli, M. A theoretical study of the lowest electronic states of azobenzene: the role of torsion coordinate in the *cis–trans* photoisomerization. *Theor. Chem. Acc.* **111,** 363–372 (2004).
3. Biswas, N. & Umapathy, S. Density functional calculations of structures, vibrational frequencies, and normal modes of *trans*- and *cis*-azobenzene. *J. Phys. Chem. A* **101,** 5555–5566 (1997).
4. Manca, C., Tanner, C., Coussan, S., Bach, A. & Leutwyler, S. H atom transfer along an ammonia chain: Tunneling and mode selectivity in 7-hydroxyquinoline·$(NH_3)_3$. *J. Chem. Phys.* **121,** 2578–2590 (2004).
5. Cusati, T., Granucci, G., Persico, M. & Spighi, G. Oscillator strength and polarization of the forbidden n→π* band of *trans*-azobenzene: A computational study. *J. Chem. Phys.* **128,** 194312 (2008).
6. Conti, I., Garavelli, M. & Orlandi, G. The different photoisomerization efficiency of azobenzene in the lowest nπ* and ππ* singlets: The role of a phantom state. *J. Am. Chem. Soc.* **130,** 5216–5230 (2008).
7. Andersson, J.-Å., Petterson, R. & Tegnér, L. Flash photolysis experiments in the vapour phase at elevated temperatures I: spectra of azobenzene and the kinetics of its thermal *cis-trans* isomerization. *J. Photochem.* **20,** 17–32 (1982).
8. Simulations have been performed using PGOPHER, a Program for Simulating Rotational Structure, C. M. Western, University of Bristol, http://pgopher.chm.bris.ac.uk.
